# Effect of Polyethylene Glycol Incorporation in Electron Transport Layer on Photovoltaic Properties of Perovskite Solar Cells

**DOI:** 10.3390/nano10091753

**Published:** 2020-09-04

**Authors:** Bo-Tau Liu, Bo-Wei Guo, Rathinam Balamurugan

**Affiliations:** Department of Chemical and Materials Engineering, National Yunlin University of Science and Technology, Yunlin 64002, Taiwan; mfhbonj@gmail.com

**Keywords:** perovskite solar cell, low temperature process, polyethylene glycol, zinc oxide, passivation

## Abstract

Due to the characteristics of high electron mobility, ambient stability, proper energy level, and low processing temperature, zinc oxide (ZnO) has become a very promising electron transport material for photovoltaics. However, perovskite solar cells fabricated with ZnO reveal low efficiency because perovskite crystals may decompose thermally on the surface of ZnO as a result of proton transfer reactions. In this study, we are the first to incorporate an inexpensive, non-toxic polyethylene glycol (PEG) into ZnO and explore the passivation effect on the electron transport layer of perovskite solar cells. Suspension stability, surface roughness, electrical conductivity, crystal size, and photovoltaic properties with respect to the PEG incorporation are analyzed. The experimental results revealed that PEG incorporation effectively passivated the surface defects of ZnO, increased the electrical conductivity, and suppressed the charge recombination. The photocurrent density could increase from 15.2 to 19.2 mA/cm^2^, an increase of 27%.

## 1. Introduction

Organic–inorganic lead halide perovskite solar cells (PSCs) have revolutionized the field of photovoltaics due to low cost, facile preparation process, high power conversion efficiency (PCE), extremely high optical absorption coefficient, and very long carrier lifetime/good stability [1,2,3,4]. The architecture of a typical perovskite solar cell is as follows: electrode/electron transport layer (ETL)/perovskite layer/hole transport layer (HTL)/electrode. In state-of-the-art perovskite solar cells, both ETL and HTL extract charge carriers from the perovskite layer efficiently, deliver the charges to the electrode, and block the opposite charge transfer [5,6,7,8]. These layers are critical for achieving high efficiency cells because they prevent severe carrier recombination at interfaces, which may dictate the open circuit voltages (V_oc_) and fill factors (FFs) of solar cells [1,9,10]. Especially, ETL plays an important role in the crystallization and morphology of perovskite film. As a result, many studies have reported on how to select or improve ETL materials, which have become a research hotspot for PSCs [11,12,13,14].

Among various ETL materials reported in the literature, TiO_2_ is the most popular and extensively studied on PSCs [5,7,15,16,17]. However, TiO_2_ has limitations such as (i) low electron mobility, which could create an unbalanced charge transport in the perovskite [18,19,20], (ii) severe J–V hysteresis phenomena owing to poor conductivity of TiO_2_ [21,22], and (iii) high-temperature sintering to obtain the desired nanostructures, which is a barrier to low cost and stretchable device fabrication [23]. ZnO is a practical alternative to TiO_2_ to the ETL of solar cells [21,24,25,26,27,28] and is also compatible to flexible and tandem devices [11] such as laser [27], detectors [27], and LED [29]. It is mainly because ZnO has similar energy levels and relatively higher electron mobility compared to TiO_2_. The low-temperature process ability, comparable energy levels, and ambient stability make ZnO an excellent electron transport material [30,31]. Moreover, the ZnO electron transport layer can be easily deposited through electrodeposition [32], atomic layer deposition [24,33], or spin-coating [34]. Such advantages cause ZnO to become very promising materials for ETL. However, ZnO has the following limitations: (i) stability of ZnO respective to perovskite film is lower than that of TiO_2_ [4,35,36,37], (ii) the deposition temperature for the perovskite layer on ZnO needs to be limited to avoid thermal decomposition (due to deprotonation of methyl ammonium ions on the ZnO surface) [36], and (iii) ZnO-based PSCs have lower efficiency compared with TiO_2_-based PSCs. The limitations lead to a low PCE of the PSC with ZnO HTL [31,38,39,40]. Normally, the PCE is below 10%, subject to the research group’s skill. In order to improve the efficiency, some passivation methods have been reported to reduce the trap states of ZnO or prevent ZnO from contacting the perovskite layer. Polyethyleneimine was used to separate ZnO from the perovskite layer as a buffer layer. It was found that the crystallization of perovskite and the PCE of solar cells were functions of the buffer layer [41]. Moreover, the surface of the ZnO layer modified by 3-aminopropanioc acid could reduce defect density and porosity of the crystalline perovskite layer, and thereby increase PCE [42]. However, the studies about how to improve the ZnO ETL for PSCs are still few. It remains a challenge to develop new passivation techniques for ZnO ETL of PSCs.

We are interested in exploring whether cheaper, non-toxic materials can be used to alter the properties of ZnO and improve the PCE of ZnO-based PSCs. Polyethylene glycol (PEG), being an inexpensive and easily accessible material, is widely used for synthesis of polyester and polyurethane. PEG has been applied as a buffer layer between HTL and the perovskite layer to improve conductivity and morphology [43] and capped with fullerene or incorporated directly into the perovskite layer to improve the PCE [44,45,46]. In the studies of bulk heterojunction polymer solar cells, PEG displayed the potential for passivating the surface of ZnO, depressing charge recombination, reducing series resistance, and improving interfacial properties [47]. The PCE of the polymer solar cells can increase by PEG interfacial engineering from 5.39 to 6.59% [48]. In this study, we are the first to attempt to incorporate PEG into the ZnO for the ETL of PSCs. We first synthesized ZnO sol by the sol–gel method. The optimal thickness (layers) of ZnO was evaluated. The PSCs with indium tin oxide (ITO)/ZnO/CH_3_NH_3_PbI_3_/Spiro-OMeTAD/Ag structure were fabricated. The effect of the PEG incorporation into the ZnO layer on photovoltaic properties was investigated. Suspension stability, surface roughness and electrical conductivity of HTL, perovskite crystal size, and photovoltaic properties with respect to the PEG incorporation were analyzed. The results revealed that incorporating PEG into ZnO ETL significantly improved short circuit current, fill factors, and PCE. At 0.2 wt% PEG incorporation, the PCE increased from 9.19 to 11.48%, an enhancement of 25%. This improvement was mainly due to the increase of the photocurrent density from 15.2 to 19.2 mA/cm^2^, an increase of 27%.

## 2. Experimental

### 2.1. Materials

ITO-coated glass substrates (7 Ω sq^−1^) were purchased from Ruilong optoelectronics (Miaoli, Taiwan). Zinc acetate dehydrate, hydrogen iodide, and PEG (MW: 20,000) were purchased from Alfa Aesar (Shanghai, China). Lead (II) iodide and lithium bis(trifluoromethane sulphonyl)imide (LiTFSI) were purchased from Acros Organics (Fukuoka, Japan). Dimethyl sulfoxide (DMSO, 99.9%) and 4-tert-butylpyridine were purchased from Sigma-Aldrich (St. Louis, MO, USA). Chloroform, chlorobenzene, dimethyl formamide (DMF, 99.9%), diethyl ether, and butanol were purchased from J.T. Baker (Phillipsburg, NJ, USA) and used as such without further purification. Toluene, ethanol, and acetonitrile were received from Echo Chemical (Maioli, Taiwan). Spiro-OMeTAD and aminomethane were purchased from Lumtec (New Taipei city, Taiwan) and Showa Chemical (Tokyo, Japan), respectively.

### 2.2. Synthesis of Methylammonium Iodide (CH_3_NH_3_I, MAI)

CH_3_NH_3_I was synthesized by dropwise addition of 10 mL of aqueous HI (57%) into 25 mL of methylamine (33%) under stirring at 0 °C. After allowing the solution to stir for two hours, the solvent was removed by rotary evaporation. The yellow–white crystals were washed three times by sonication in diethyl ether, filtered, and dried at 60 °C overnight under vacuum to yield white crystals of CH_3_NH_3_I [49].

### 2.3. Synthesis of Zinc Oxide

Zinc oxide precursor was synthesized as reported elsewhere [50]. First, 2.95 g of anhydrous zinc acetate was added to 125 mL of methanol at 65 °C, and then 1.48 g of potassium hydroxide was added to 65 mL of methanol solution at room temperature. The zinc acetate solution was slowly dropped into the potassium hydroxide solution for at least 15 min, and the reaction was carried out for 2.5 h. After that, the resulting white turbid suspension was washed two times with methanol through centrifugation (1500 rpm for 10 min). The precipitate was re-dispersed into the mixture of n-butanol, methanol, and chloroform in the volume ratio of 14:1:1, resulting in ZnO solution (6 mg/mL). As-prepared ZnO solution was mixed with various PEG contents (0, 0.1, 0.2, and 0.3 wt%).

### 2.4. Device Fabrication

**Figure 1:**
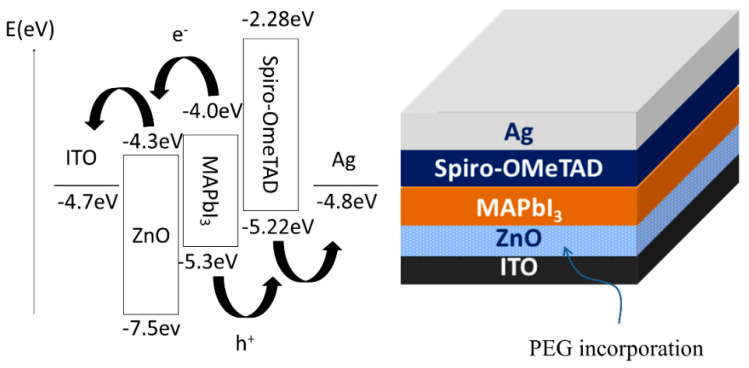
The schematic representation of energy diagram as well as structure of designed perovskite solar cells.

The ITO glass substrates were masked by PI tape in a T-shaped pattern (2 × 1.5 cm), and then the unmasked portion of the ITO was etched with zinc powder and HCl solution (6 M). After etching, the ITO glass was cleaned with a detergent to remove the surface residue and zinc powder, and then ultrasonically with ethanol, isopropyl alcohol, and deionized water for 15 min, respectively. After cleaning, the substrates were blow-dried with nitrogen and then treated by the plasma cleaner (PDC-23G, Harrick Plasma, Ithaca, NY, USA) for 10 min. The ZnO solution with various PEG contents was spin-coated on the cleaned ITO glass substrates at 3000 rpm for 30 s and then heated at 125 °C for 30 min. CH_3_NH_3_PbI_3_ (MAPbI_3_) was prepared by dissolving MAI and PbI_2_ in DMF in a ratio of 1:1 and then stirring the solution for one day. The 40-wt% MAPbI_3_ solution was spin-coated on the prepared ZnO-PEG substrates at 5000 rpm for 30 s in a glove box (Younme, Taoyuan, Taiwan). After spinning for 4 s, 105 μL of toluene was dropped upon the MAPbI_3_ coated substrates as an anti-solvent. The substrates were then heated at 100 °C for 5 min. Eighty milligrams of Spiro-OMeTAD, 17.5 μL of Li-TFSI (520 mg/mL acetonitrile solution), and 28.5 uL of 4-TBP were mixed into 1 mL of chlorobenzene. The solution was spin-coated on the perovskite layer at 2000 rpm for 30 s. Finally, a silver layer with 100-nm thickness was formed on the surface of the devices through thermal deposition (thermal evaporator, Kao-Duen Technology, New Taipei city, Taiwan) under a vacuum of 5 × 10^−6^ torr and a plating rate of 0.8–0.9 Å/s. The structure of the devices is shown in Figure 1. ZnO revealed a suitable energy level for MAPbI_3_ and ITO.

### 2.5. Measurements and Characterization

The profile and conductivity of the ZnO layer were measured using a scanning probe microscope (SPM, Dimension ICON, Bruker, Billerica, MA, USA) and a four-pin probe meter (Loresta-GP, Mitsubishi Chemical, Tokyo, Japan) with an MCP-T610 probe, respectively. The morphologies of the MAPbI_3_ layer and the devices were examined using a field-emission scanning electron microscope (JSM-7401F, JEOL, Tokyo, Japan). Photoluminescence (PL) spectra of the perovskite layer coated upon various ZnO layers were measured using a fluorescence spectrophotometer (LS-55/45, PerkinElmer, Waltham, MA, USA). The photocurrent density–voltage (J–V) characteristics were measured under irradiation of 100 mW·cm^−2^ using a solar simulator (MFS-PV, Hong-Ming Technology, New Taipei city, Taiwan) equipped with a source meter (Keithley 2400, Keithley Instruments, Solo, OH, USA). Electrochemical impedance spectra (EIS) were measured over the frequency range of 50 mHz to 100 kHz with a potential perturbation of 10 mV using an electrochemical workstation (Zennium, Zahner, Kronach, Germany).

## 3. Results and Discussion

**Figure 2:**
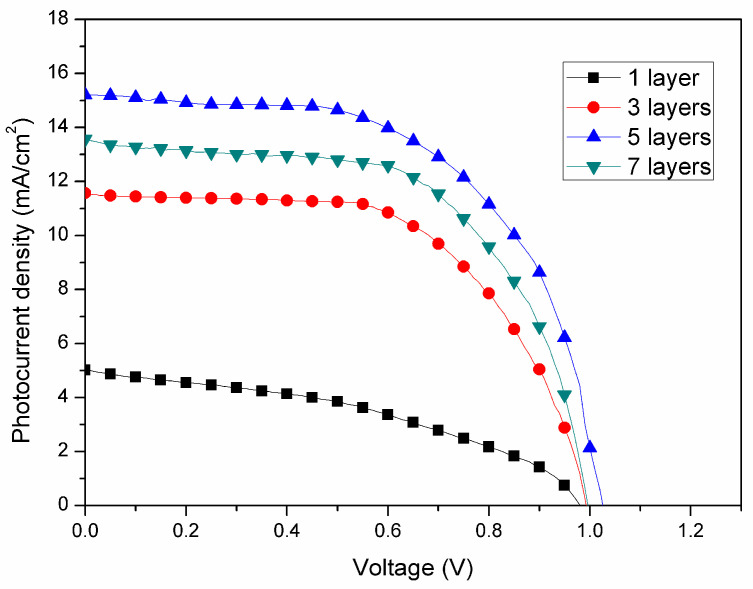
Photocurrent–voltage curves of perovskite solar cells for different layers of ZnO without PEG-incorporation as the electron transport layer.

**Figure 3:**
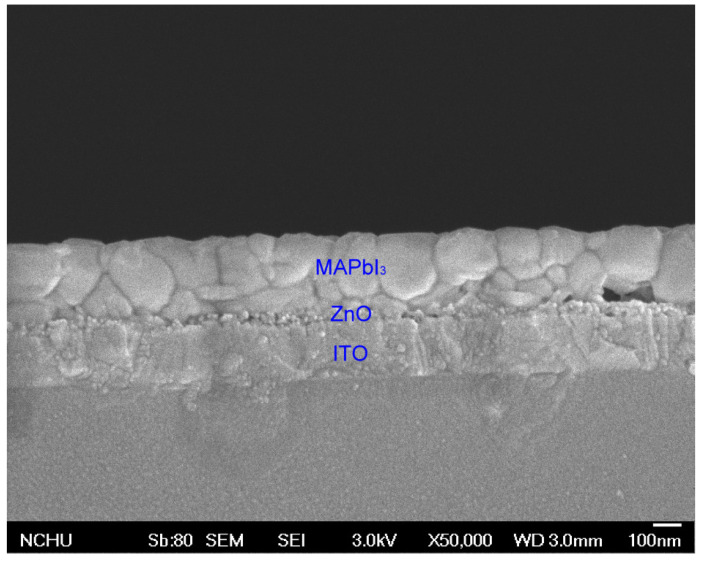
Scanning electron microscopic image of ITO/ZnO/MAPbI_3_.

Zinc oxide has good carrier selectivity, which can separate electrons from holes and serve as an electron transport layer. To evaluate the effect of thickness of ZnO layer on photovoltaic performance, devices were prepared by spin coating various amounts of ZnO solution (one, three, five, and seven layers), where the thickness of each ZnO layer was near 6 nm. The J–V curves of the PSCs with various thicknesses of ZnO layers without PEG incorporation are shown in Figure 2. The corresponding photovoltaic characteristics are summarized in Table 1. It can be seen that the PCE of PSCs increased with increasing the coating number of the ZnO layer, reached a maximum at five layers, and decreased with a further increase of the coating number. Generally, the increase of thickness of HTL increases the distance of electron transport from the perovskite layer to the ITO. The longer electron diffusion path raises the probability of electron–hole recombination, decreasing the current density and thereby the efficiency [51,52]. However, too thin HTL may lead to incomplete coverage on the surface of the ITO layer and make the perovskite layer contact the ITO layer directly or insufficiently facilitate photo-generated electrons to the ITO layer and block photo-generated holes generated from the perovskite layer. In our experiments, coating five layers achieved an optimal thickness (~30 nm, as shown in Figure 3), revealing the best efficiency and featuring highest short-circuit current as well. As a result, we prepared PSCs with five-layer ZnO for the further study of the effect of PEG incorporation in the ZnO layer on photovoltaic properties.

**Figure 4:**
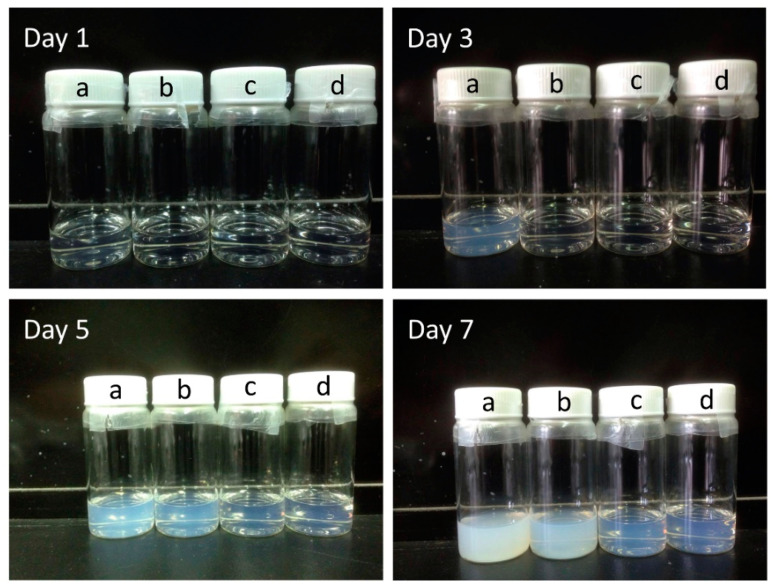
Photo images for suspension stability of ZnO and ZnO-PEG solutions at different elapsed time (**a**: ZnO, **b**: ZnO-PEG1, **c**: ZnO-PEG2, and **d**: ZnO-PEG3).

In order to realize the effect of PEG on ZnO, we recorded the variation of the stability of the ZnO solution with various PEG contents (Figure 4). It was observed that the as-prepared ZnO solution became a white turbid liquid gradually after standing for a few days, indicating that ZnO nanoparticles aggregated to form large particles over a period of time. However, the suspension became more stable after the addition of PEG. The suspension stability increased with increasing PEG content. The result may be due to the strong interaction between the hydrophilic ether groups/hydroxyl groups of PEG and the polar surface of ZnO nanoparticles. The adsorption of PEG on the surface of ZnO nanoparticles may reduce aggregation [53].

**Figure 5:**
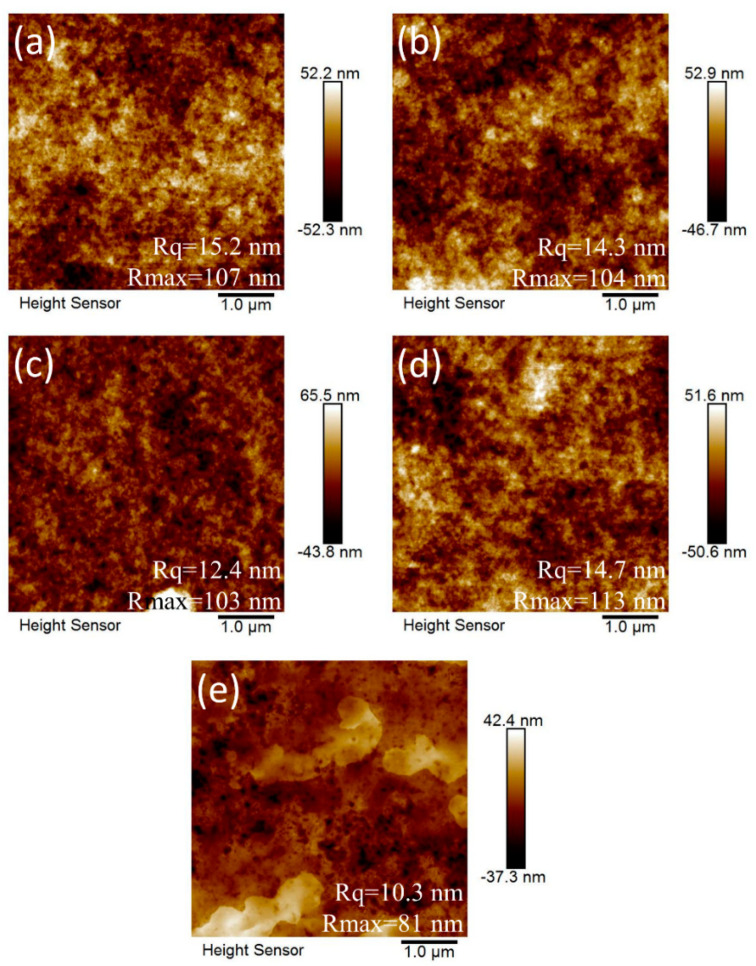
Scanning electron microscopic images of ZnO and PEG-incorporated ZnO layers: (**a**) pristine ZnO, (**b**) ZnO-PEG1, (**c**) ZnO-PEG2, (**d**) ZnO-PEG3, (**e**) ZnO/PEG1.

The morphologies of the coating layers made from the PEG modified ZnO nanoparticles were studied by SPM (Figure 5). With increasing PEG content, root-mean-square (RMS) surface roughness (Rq) of the ZnO layer decreased from 15.2 to 12.4 nm. The result implies that increasing the suspension stability is helpful to form a smoother coating surface. However, too much PEG may hinder the coating from flowing resulting in the Rq of ZnO-PEG3 rising to 14.7 nm. If a 1 wt% PEG solution is spin-coated on the ZnO layer (ZnO/PEG1), the surface roughness is only 10.3 nm due to the height difference of the ZnO surface filled by PEG to make the surface smooth.

**Figure 6:**
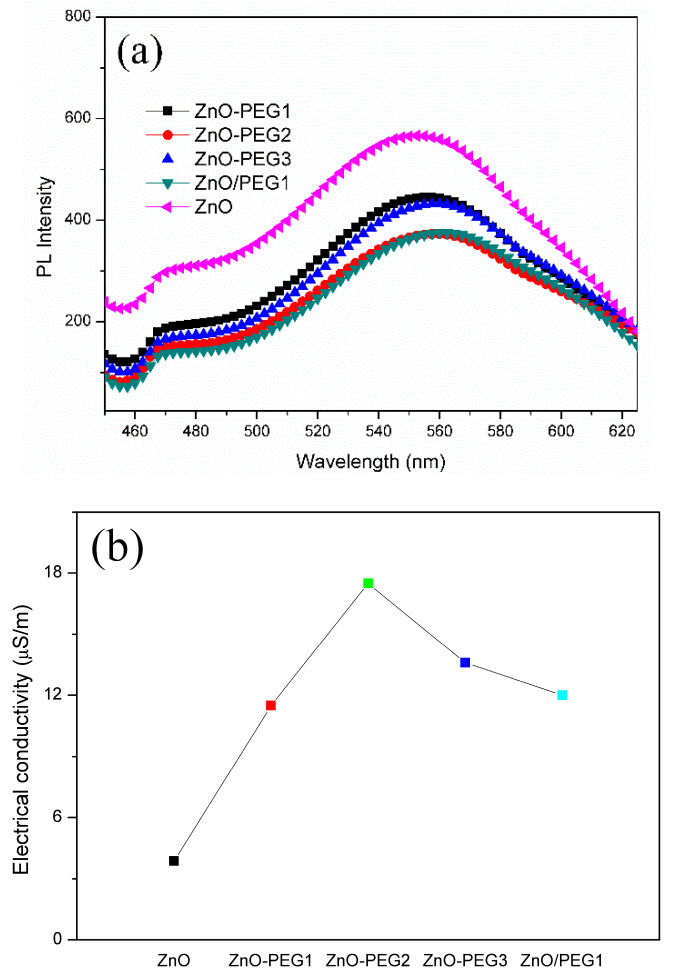
(**a**) Photoluminescence spectra under 365-nm excitation and (**b**) electrical conductivities of pristine and PEG-incorporated 5-layer ZnO.

Figure 6a shows the PL spectra of the pristine ZnO and the PEG modified ZnO samples. The ZnO exhibited strong green emission near 550 nm due to the radiative recombination of a photo-generated hole with an electron attributed to an oxygen vacancy and the presence of surface defects on the nanoparticles [54,55]. When the ZnO was incorporated with PEG, the PL emission caused by the charge recombination was suppressed. The suppression of PL emission was also observed for the case of a PEG layer over the ZnO layer (PEG/ZnO). It was found that the PL intensity decreased with the surface roughness. The results imply that PEG may fill up the surface defects of ZnO to alleviate the recombination of electrons and holes. In addition to that, the PEG incorporation also increased the electrical conductivity of ZnO, which rose from 3.87 × 10^−6^ to 1.75 × 10^−5^ S/m, a near five-fold enhancement (Figure 6b). It reduces electrons being trapped in surface defects so that the lone pair of electrons on the oxygen atom of polyethylene glycol may coordinate with zinc oxide. However, too much PEG incorporation hinders the charge transport among ZnO nanoparticles (ZnO-PEG3).

**Figure 7:**
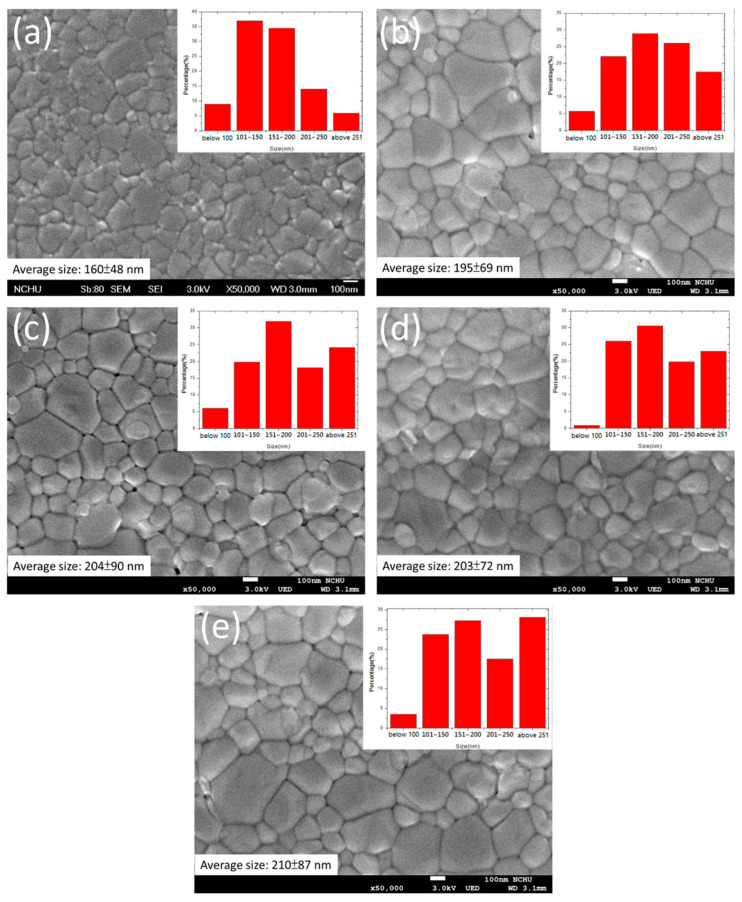
Scanning electron microscopic images of MAPbI_3_ layers coated on 5-layer ZnO with various PEG incorporation: (**a**) ZnO, (**b**) ZnO-PEG1, (**c**) ZnO-PEG2, (**d**) ZnO-PEG3, and (**e**) ZnO/PEG1.

**Figure 8:**
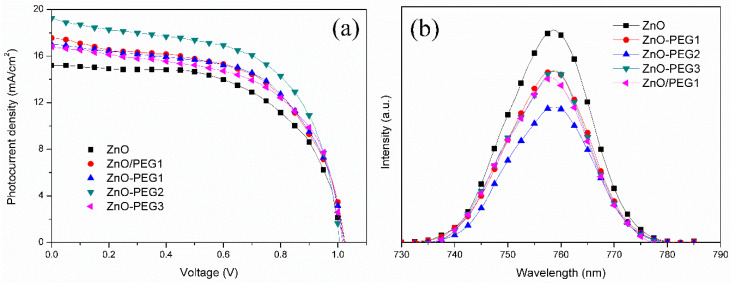
(**a**) Photocurrent–voltage curves of perovskite solar cells and (**b**) photoluminescence spectra of MAPbI_3_ films under 385-nm excitation with pristine or PEG-incorporated 5-layer ZnO as the electron transport layer.

**Figure 9:**
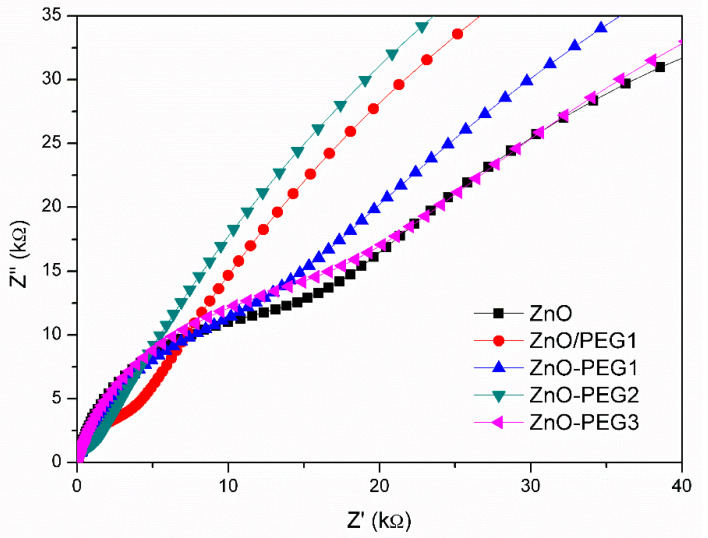
Nyquist curves of perovskite solar cells with pristine or PEG-incorporated 5-layer ZnO as the electron transport layer.

When the MAPbI_3_ solution was coated on the ZnO layer, the perovskite crystal grew with different sizes over various PEG-incorporating ZnO (Figure 7). The MAPbI_3_ crystal became noticeably larger for the cases of the presence of PEG in ZnO. However, all the PEG concentrations (0.1, 0.2, 0.3, or 1 wt% coating) resulted in similar perovskite crystal sizes. The result reveals that PEG is in favor of the MAPbI_3_ growth. Figure 8a shows the current density–voltage (*J*–*V*) curves of the PSCs with architectures of ITO/ZnO-PEG/MAPbI_3_/Spiro-OMeTAD/Ag, and the corresponding photovoltaic characteristics are listed in Table 2. Compared with pure ZnO, the PEG-incorporation increased photocurrent density from 15.2 to 19.2 mA/cm^2^ (ZnO-PEG2), an increase of 27%. The PL intensity of the ITO/ZnO-PEG/MAPbI_3_ decreased with increasing PEG content. ZnO-PEG2 had the lowest intensity (Figure 8b). As seen in Figure 8, the photocurrent density increased with decreasing PL intensity. The result implies that the decrease of the PL intensity is ascribed to the drop in the rate of charge recombination, thus increasing photocurrent density. However, too high PEG content or covering a PEG layer makes the PL intensity increase, which decreases photocurrent density and PCE. As a result, the PL intensity is against the PCE, which implies that the charge recombination dominates the efficiency. The PSCs were subjected to EIS analysis to further investigate their charge carrier transport properties systematically. As shown in Figure 9, the Nyquist plots of the EIS exhibited two semicircles for various PEG contents. The semicircle in the high-frequency region was associated with the charge transfer resistance (R2), whereas the semicircle in the low-frequency region was related to the charge recombination resistance (R3). The corresponding resistances for PSCs with various PEG incorporations in five-layer ZnO are listed in Table 3. With the increase of PEG incorporation (ZnO-PEG1, ZnO-PEG2), the transfer resistance decreased but the charge recombination increased. According to morphology and conductivity analysis (Figure 5 and Figure 6b), the decrease of charge transport resistance and suppression of charge recombination may be attributed to the increase in the electrical conductivity of the ETL and the increase in the crystal size of MAPbI_3_. Too much PEG incorporation (ZnO-PEG3) covering a PEG layer on the ZnO ETL (ZnO/PEG1) may lead to the increase of R2 and the decrease of R3, which may result from the decrease of the electrical conductivity of the ETL and the increase of the distance of charge transport.

## 4. Conclusions

In summary, we are the first to attempt to incorporate an inexpensive, nontoxic PEG into the ZnO for the electron transport layer of PSCs. The passivation effect of PEG on the photovoltaic properties of ZnO was analyzed in detail. Except for stabilizing the ZnO solution, PEG incorporation can passivate the surface defects of ZnO to increase the electrical conductivity from 3.87 × 10^−6^ to 1.75 × 10^−5^ S/m and reduce the surface roughness of the ZnO layer from 15.2 to 12.4 nm. Moreover, the PEG-incorporated ZnO layer helps the MAPbI_3_ crystal grow larger. The PSC with 0.2 wt% PEG incorporation showed the increase of the photocurrent density from 15.2 to 19.2 mA/cm^2^ and the highest PCE of 11.48% (an enhancement of 25%). The PCE and photocurrent enhancement may arise from the decrease on both charge transfer resistance and rate of charge recombination due to the high electrical conductivity and large crystal size caused by PEG incorporation.

## Figures and Tables

**Table 1:** Photovoltaic characteristics of perovskite solar cells with different layers of ZnO.

No. of ZnO Layer	Voc (V)	Jsc (mA/cm^2^)	FF (%)	η (%)
1	0.98	5.0	41.0	2.0
3	0.99	11.6	59.5	6.8
5	1.02	15.2	59.3	9.2
7	0.99	13.6	60.1	8.1

**Table 2:** Photovoltaic characteristics of perovskite solar cells incorporating various PEG amounts in the ZnO layer.

Sample	PEG content (wt%)	Voc (V)	Jsc (mA/cm^2^)	FF (%)	η (STD ^c^) (%)
ZnO	0 ^a^	1.02	15.2	59.3	9.2 (0.21)
ZnO-PEG1	0.1 ^a^	1.02	16.6	61.5	10.0 (0.30)
ZnO-PEG2	0.2 ^a^	1.01	19.2	59.4	11.5 (0.12)
ZnO-PEG3	0.3 ^a^	1.02	16.5	59.4	10.4 (0.21)
ZnO/PEG1	1^b^	1.02	17.6	59.6	10.7 (0.25)

^a^ the weight ratio of PEG to ZnO. ^b^ 1 wt% PEG in chlorobenzene was coated upon the ZnO layer. ^c^ standard deviation calculated from 10 cells.

**Table 3:** Electrochemical impedance analysis of perovskite solar cells incorporating various PEG amounts in the ZnO layer.

Sample	R_1_ (Ω)	R_2_ (kΩ)	R_3_ (kΩ)
ZnO	36.8	14.0	104
ZnO-PEG1	35.1	8.5	177.9
ZnO-PEG2	34.0	0.81	205.7
ZnO-PEG3	35.1	15.1	131.8
ZnO/PEG1	34.1	2.7	179.1
